# Pseudohypoparathyroidism Presenting With Recurrent Twitching: Challenges Making a Diagnosis in a Low-Resource Environment

**DOI:** 10.1155/crie/5272560

**Published:** 2025-11-21

**Authors:** Phoebe Wamalwa, Prisca Amolo

**Affiliations:** ^1^Department of Paediatrics, Kenyatta University, Nairobi, Kenya; ^2^Department of Paediatrics, Kenyatta National Hospital, Nairobi, Kenya

## Abstract

Pseudohypoparathyroidism (PHP) is a metabolic disorder that occurs due to target end-organ resistance to parathyroid hormone (PTH). It is a rare cause of severe symptomatic hypocalcemia as it characteristically manifests with high phosphate and low calcium. The clinical presentation, biochemical features, and severity vary from patient to patient leading to delay in diagnosis. Reduced awareness and lack of recognition of this rare clinical syndrome coupled with limited resources in rural health facilities also contribute to missed or late diagnosis. Reported here is a case of a 13-year-old girl who presented in a rural county hospital with a 1-year duration of muscle twitching and persistent headache. She had been managed with anticonvulsant therapy without resolution. Upon admission to our facility calcium levels were noted to be very low with high phosphate, high PTH levels, and normal vitamin D levels. The brain CT scan revealed calcifications in the basal ganglia. A diagnosis of PHP was henceforth made. She was put on intravenous calcium gluconate with subsequent oral calcium and calcitriol with resultant resolution of twitching. This case points to delayed diagnosis of a rare cause of symptomatic hypocalcemia signifying importance of early biochemistry testing and careful interpretation in a patient presenting with persistent twitching in low-resource set ups.

## 1. Introduction

Hypocalcemia is a common metabolic cause of convulsions in the pediatric population, with severity ranging from mild asymptomatic states to severe, life-threatening episodes [1]. Calcium levels are tightly regulated within a narrow range of 2.1–2.6 mmol/L by three hormones, vitamin D, parathyroid hormone (PTH), and calcitonin [1, 2].

Activated vitamin D enhances intestinal absorption of calcium and phosphate, maintaining mineral balance [3]. PTH, secreted by the parathyroid glands, is the principal regulator of calcium homeostasis. PTH enhances calcium reabsorption in the distal convoluted tubule and reduces phosphate reabsorption in the proximal tubule, while also stimulating the activation of vitamin D [4]. Calcitonin counteracts hypercalcemia by inhibiting osteoclastic bone resorption but has a minimal physiological role under normal conditions [5].

Hypocalcemia most often results from vitamin D deficiency [1]. However, in children, low or inappropriately normal PTH levels suggest hypoparathyroidism as an important differential diagnosis. Causes of hypoparathyroidism include genetic mutations, autoimmune destruction, postsurgical or postirradiation injury to the parathyroid glands, and hypomagnesemia [6]. In contrast, secondary hypocalcemia with elevated PTH may arise from chronic renal disease, vitamin D deficiency, or resistance to PTH action (pseudohypoparathyroidism [PHP]). Less common causes of low calcium include drugs like bisphosphonates and diuretics [7], critical illness, or hypermagnesemia [2]. Differentiating these entities is essential, as management varies according to the underlying mechanism.

PHP is a rare cause of hypocalcemia with an estimated prevalence of 0.3–1.1 in 100,000 depending on the geographical zone [8]. PHP is an inherited disorder characterized by unresponsiveness of end-organ to the PTH [9, 10]. The biochemical findings and symptomatology therefore resemble hypoparathyroidism with hypocalcemia and hyperphosphatemia but instead of low PTH levels the patients usually have high PTH levels [8] with or without physical features of Albright hereditary osteodystrophy (AHO). PHP is generally classified into PHP-1a and PHP-1b. Broadly, there is a blunted response of cAMP and excretion of phosphate in urine following activation of G-protein-coupled receptor for PTH in the downward cascade [11]. PHP-1b is isolated resistance to PTH in the renal tubules but is presumed to always have normal PTH response in bone [11]. Therefore, patients typically lack phenotypic features of AHO. However, AHO features have been reported in rare PHP1b cases, suggesting clinical overlap among PHP disorders despite different molecular defects. Some hormone resistance, especially to thyroid-stimulating hormone (TSH), is observed in patients with PHP type 1b. This subtype is associated with deletions in the STX16 gene that lead to loss of methylation at the GNAS A/B differentially methylated region (DMR), resulting in maternally inherited autosomal dominant PHP type 1B [10, 12]. However, previous studies indicated no significant difference in the presence or severity of TSH resistance between familial PHP1b cases with STX16 deletions and those caused by other epigenetic or imprinting abnormalities [8]. This suggests that TSH resistance reflects a broader GNAS-related signaling defect rather than a specific genotype effect. Most PHP-1b cases are sporadic, arising from epigenetic or structural changes in the upstream GNAS region that lead to complex methylation abnormalities distinct from the STX16-related autosomal dominant subtype [10, 12].

PHP-1a has PTH resistance in both kidneys and bone and expresses a phenotype of AHO [13, 14]. Patients with AHO phenotype express short stature, round face, obesity, short metacarpals, presenting with lack of knuckles at the third, fourth, or fifth metacarpals [14, 15]. These patients may have reduced intelligence, short neck, and subcutaneous ossifications [16]. PHP-1a has reduced Gs alpha activity, while PHP-1b has normal Gs activity in erythrocytes. PHP-1a has multiple organ resistance, including TSH, follicle-stimulating hormone (FSH), and luteinizing hormone (LH) that act via G-protein-coupled receptor. G-protein is a multimeric complex that includes G-protein alpha-subunit (Gsα) encoded by *GNAS1* gene. When a loss-of-function mutation affects the maternal allele, the G-protein complex cannot act properly in some tissues where the imprinting is localized. There is, however, no GH resistance in PHP-1a [17, 18], which does not mediate its actions through a G-protein-coupled receptor. There is little to no evidence of resistance to LH and FSH in PHP1B. A summary of the distinguishing features between PHP-1a and PHP-1b is presented in Table 1.

In the Consensus Statement, 2018, the participants agreed that the diagnosis of PHP should be based on major criteria, including resistance to PTH, ectopic ossifications, brachydactyly, and early-onset obesity [9]. This should henceforth be followed by a confirmatory molecular genetic analysis. Screening of patients at diagnosis and during follow-up for specific multihormone features, such as PTH resistance, TSH resistance, growth hormone deficiency, hypogonadism, skeletal deformities, oral health, weight gain, glucose intolerance or type 2 diabetes mellitus, and hypertension was recommended [9]. Additionally, organ ossifications and neurocognitive impairment need to be checked during follow-up [9]. A multidisciplinary team is therefore required for the holistic management of this condition. However, despite these recommendations, resource-constrained set-ups face a myriad of problems, including but not limited to access to not just baseline biochemistry tests but also confirmatory ones like genetic testing; scarce specialized human resources and paucity of comprehensive multidisciplinary teams in holistic management. A specific diagnosis is therefore often delayed due to a lack of recognition of the syndrome and associated biochemical and phenotypic features.

## 2. Case Report

A 13-year-old girl from a nonconsanguineous marriage presented with a 1-year history of right upper and left lower limb twitching and headache. The twitching was reportedly increasing in frequency over time, with several episodes occurring in a day. Each episode was reported to last ~5 min and was accompanied with facial mouth deviation. There was no associated mouth frothing, loss of consciousness, incontinence, or postictal sleep. The headache was one-sided and was accompanied with dizziness on and off throughout the year. She reported recurrent pharyngotonsillitis with nasal obstruction and otalgia. The birth and early developmental history were unremarkable, with no prior significant illnesses or treatments before the onset of twitching. She is the third born in a family of four. The rest of the siblings, two girls and one boy, are normal. The mother and maternal grandmother have no history of twitching or significant medical illness but are obese, with BMIs of 33.4 and 31, and heights at the 25th and 50th percentiles, respectively.

**Figure 1:**
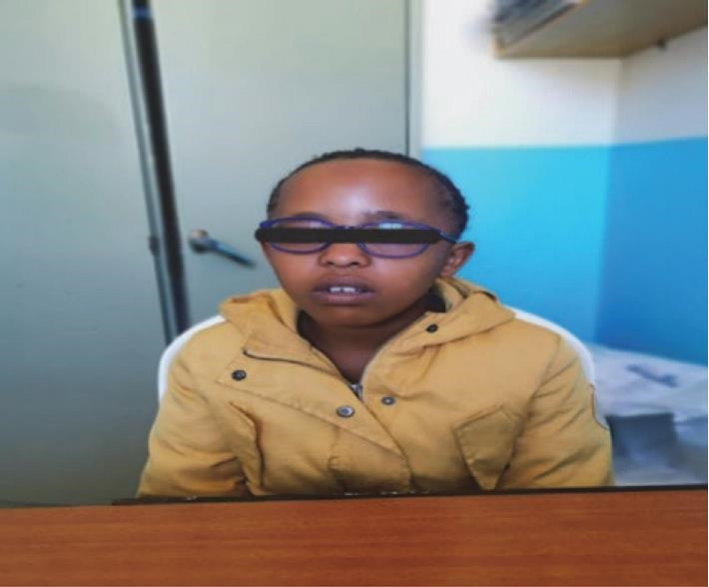
Image of case showing round face.

**Figure 2:**
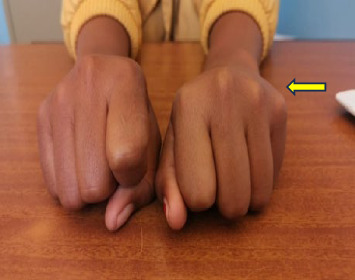
There was no dimpling of the metacarpals (yellow arrow).

Examination findings revealed an alert, short girl in good general condition with eye glasses and a relatively round face (Figure 1). There was no obvious dimpling of the third and fourth metacarpals, otherwise referred to as “knuckle, knuckle, dimple, dimple sign” (Figure 2). The metatarsals were likewise normal. There were no additional obvious abnormal skeletal findings nor subcutaneous calcifications. The rest of the systems were normal. Her intellectual capacity was average. This was inferred from school performance, as reported, and not from a standardized assessment. She was able to walk normally without any gait disturbances.

She had a weight of 47.2 kg (0 SD) and height of 143 cm (−2 SD) with a BMI of 23.1 (+1 SD) centile. The mother's height was 159 cm (−0.67 SD) with a weight of 84.5 kg (+2 SD), BMI of 33.4 (+2.68 SD).

Initial blood chemistry tests showed a corrected calcium of 0.9 mmol/L (2.02–2.6), alkaline phosphatase (ALP) of 1143 µ/L (64–306), magnesium mmol/L of 0.61 mmol/L (0.8–1), phosphate of 2.89 mmol/L (0.81–1.62), and PTH of 329.7 pg/mL (16–65). The mother's calcium and phosphate were normal. We were not able to analyze blood parameters for all the family members because of financial constraints. The thyroid, liver, and kidney function tests, Table 2, as well as the full blood count, were normal.

**Figure 3:**
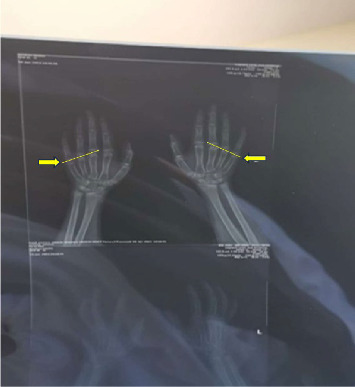
Hand radiograph showing slightly shortened 4th and 5th metacarpals as shown by yellow straight lines intersecting the 3rd metacarpal and dense sclerotic epiphyseal and metaphyseal bands, yellow arrows.

The chemistry tests were measured using an automated clinical chemistry analyzer (Abbott) while hormonal (TSH and PTH) levels were determined by chemiluminescent immunoassay on an automated immunoassay platform (Abbott Architect i2000SR). All assays were performed according to the manufacturers' instructions, and internal quality control measures were within acceptable ranges during testing. The hand radiograph revealed mild shortening of the 4^th^ and 5^th^ metacarpal (Figure 3).

**Figure 4:**
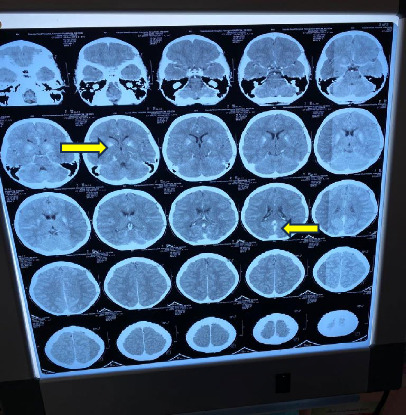
CT scan of the brain showing basal ganglia calcifications: yellow arrows.

The CT scan of the brain showed basal ganglia calcifications (Figure 4). The renal ultrasound did not show any calcifications.

The genetic testing to differentiate the subtypes was not done due to financial constraints.

The patient was given intravenous calcium gluconate followed by calcium at 50 mg/kg/day in two divided doses as well as calcitriol at 0.25 µg twice daily. However, the slightly decreased magnesium did not warrant correction, and it corrected itself. The twitching has reduced significantly to once a month, and currently she has not had any episode in the last 5 months of follow-up. The headache subsided 2 months after initiating treatment. She still gets reviewed in the ear, nose, and throat clinic for nasal blockage, but otalgia has resolved. Biochemical tests have shown improvement though 4 months after therapy initiation. Calcium levels are slightly below normal recommended levels, despite the dose being increased from the initial 50–80 mg/kg/day. The phosphate levels are still slightly raised, and PTH levels have remained high. Evaluation of hypercalciuria was not done due to financial incapacity. The ALP levels have shown a downward trend and have since normalized. The IGF-1 levels are below the normal range. Hypogonadism was not evaluated biochemically because of limited resources. However, she had already attained thelarche and menarche. Subsequent chemistry analyses are as shown in Table 1. The most recent PTH, however, seems to be rising. This rise was attributed to the reported noncompliance for 2 weeks.

## 3. Discussion

PHP is a rare metabolic disorder requiring basic knowledge in the identification of specific biochemical and clinical features in patients presenting with low calcium levels, high phosphate, high PTH levels, and organ ossifications with or without twitching and should form part of the differential diagnoses in these individuals. Early identification prevents future complications like neurocognitive impairment from brain calcifications emanating from prolonged hypocalcemia and hyperphosphatemia [19] and presents an opportunity to address related multihormone resistance with accompanying future complications. Patients from poorly resourced set ups often face delays in diagnosis and suboptimal management as a result of scarcity in appropriately trained human resources as well as inadequate diagnostics. Our patient was managed for twitching using anticonvulsant medications for over a year before bone biochemistry tests were requested. Upon admission to our facility, a brain CT scan showing calcifications in the basal ganglia, coupled with hypocalcemia, raised a high suspicion of hypoparathyroidism. However, raised PTH levels instead prompted us to make a diagnosis of PHP. She was short with a round face, and the radiological tests revealed slightly shortened 4^th^ and 5^th^ metacarpals. Her mother did not have biochemical or skeletal abnormalities, but she was obese. We would have liked to perform a confirmatory genetic test, but limited resources were an impediment. The Initiation of intravenous calcium gluconate followed by oral calcium and calcitriol resulted in cessation of twitching, and the patient has been free of twitching for over 8 months now. However, her infrequent clinic attendance and noncompliance may have contributed to suboptimal response. Otalgia and recurrent sensation of pain at the throat also resolved. This could perhaps have been due to low calcium levels causing muscle spasms and pain. However, the nasal blockage may have been a coincidence since it persisted despite replacement with calcium. Our patient underwent tests to rule out TSH resistance, and so far, they have been normal without any clinical symptoms of hypothyroidism. Her pubertal status is at tanner stage 2. The patients may show resistance to GHRH, and this was evident by low IGF-1 levels and short stature. Growth hormone stimulation test was not done owing to resource limitations. Elevated ALP in PHP-1a is unusual and is more often reported in PHP type 1b, where renal tubular resistance to PTH predominates without the full spectrum of AHO features. The elevated ALP in this case likely reflects heightened bone turnover secondary to chronic hypocalcemia rather than a hepatic etiology since the liver function tests were normal [20]. Future plans are to monitor the biochemical and clinical features of multiple hormone resistance, including stimulated GH. Evaluation of hypercalciuria, which was not done in this case due to financial incapacity, is recommended for monitoring in future management. A detailed family history investigation, including the mother's siblings' children and the grandmother's siblings, to facilitate early diagnosis of PHP, was not done, but is recommended. In the absence of confirmatory genetic testing, like in this case, other potential causes of hypocalcemia with elevated PTH must be considered. These include PHP, secondary hyperparathyroidism due to chronic vitamin D deficiency or renal dysfunction, and rare PTH receptor (PTH1R) mutations that can mimic PHP phenotypes. PTH1R mutations have been reported in conditions such as Blomstrand chondrodysplasia and Eiken syndrome, which may present with variable skeletal findings and end-organ PTH resistance [21].

Nevertheless, the constellation of hypocalcemia, hyperphosphatemia, and elevated PTH with normal renal function strongly supports PHP type 1B as the most likely diagnosis in this context.

## 4. Conclusion

Diagnosing rare endocrine diseases like PHP is particularly challenging in low-resource settings where genetic testing and specialized assays are unavailable, often leading to underdiagnosis. This case highlights delayed recognition of a rare cause of symptomatic hypocalcemia and emphasizes early and careful interpretation of basic biochemical tests (P, Mg, ALP), endocrine tests (PTH, TSH, fT4, active vitamin D), and imaging tests (bone X-ray, brain CT), to investigate the cause. Strengthening context-appropriate diagnostic algorithms and fostering international collaborations are essential to improve recognition and management of rare diseases in such environments [22].

## Figures and Tables

**Table 1:** Summary of differences between pseudohypoparathyroidism type 1a and type 1b.

Feature	PHP-1a	PHP-1b
Primary PTH resistance [11]	Kidney and bone	Kidney only (renal tubules)
Phenotype (Albright hereditary osteodystrophy, AHO) [13–15]	Present—short stature, round face, obesity, brachydactyly (short metacarpals with absent knuckles), subcutaneous ossifications, short neck	Absent in most cases; rarely present (clinical overlap reported)
Neurocognitive effects [16]	May have reduced intelligence and developmental delay	Usually, normal intelligence
Other hormone resistance [17, 18]	Frequently TSH, FSH, LH resistance (multihormone resistance via G-protein-coupled receptors); no GH resistance	Sometimes mild TSH resistance; little or no evidence of LH/FSH resistance
Gsα activity in erythrocytes [17, 18]	Reduced	Normal
Genetic defect [10, 12]	Loss-of-function mutations in maternal GNAS1 allele (affecting Gsα)	Most commonly due to STX16 gene deletions (autosomal dominant forms) or complex GNAS methylation abnormalities (sporadic)
Inheritance [12]	Autosomal dominant with imprinting effect (maternal allele)	Sporadic or autosomal dominant (STX16 deletion)

**Table 2:** Bone chemistry and hormonal analyses trends during follow-up of the case.

Date	Calcium (corrected) (mmoL/L)	Phosphate (mmol/L)	Magnesium (mmol/L)	ALP (U/L)	PTH	Vit D (ng/mL)	IGF-1 levels (ng/mL)	Urea (mmol/L)	TSH (mIU/L)	Creatinine (umol/L)
Baseline	**0.9** (2.2–2.7)	**2.89** (1.33–1.92)	**0.56** (0.65–1.05)	**1143** (30–180)	**329.7** pg/mL (15–68)	**40** (30–100)		**4.07 (1.7–8.3)**	**2.05 (0.7–4.17)**	**57.6 (53–97)**

2 weeks	**1.9**		**0.61**							

2 months	**1.1**			**820** (64–306)						

3 months	**1.1**	**2.46**	**0.91**	**700** (64–306)						

3.5 months	**1.5**	**1.77**	**1.01**	**883** (64–306)						

4 months	**1.9**	**2.04**			**78.4** pmol/L (1.3–9.3 pmol/L)					

5 months	**1.35**	**1.76**		290 (62–280)			**107** (170–527)			

8 months	**1.68**	**2.22**		267 (62–280)						

*Note:* Bold values indicate the patient's measured results in comparison with the corresponding reference ranges shown in brackets.

## Data Availability

The data that support the findings of this study are available upon request from the corresponding author. The data are not publicly available due to privacy or ethical restrictions.

## General Statement

The genetic nor epigenetic testing was not done due to financial constraints. This is critical for proper diagnosis of PHP1a/1b. Since PHP1b patients occasionally show mild AHO features, it remains unclear whether this case is 1a or 1b. Screening of family members for parameters in basic blood chemistry is useful but was not done due to financial indisposition. Evaluation of hypercalciuria was also not done but this is recommended for monitoring during management.
